# Sex Matters: Physiological Abundance of Immuno-Regulatory CD71+ Erythroid Cells Impair Immunity in Females

**DOI:** 10.3389/fimmu.2021.705197

**Published:** 2021-07-21

**Authors:** Siavash Mashhouri, Petya Koleva, Mai Huynh, Isobel Okoye, Shima Shahbaz, Shokrollah Elahi

**Affiliations:** ^1^ School of Dentistry, Division of Foundational Sciences, Faculty of Medicine and Dentistry, University of Alberta, Edmonton, AB, Canada; ^2^ Department of Oncology, Faculty of Medicine and Dentistry, University of Alberta, Edmonton, AB, Canada; ^3^ Department of Medical Microbiology and Immunology, Faculty of Medicine and Dentistry, University of Alberta, Edmonton, AB, Canada; ^4^ Li Ka Shing Institute of Virology, University of Alberta, Edmonton, AB, Canada

**Keywords:** CD71+ erythroid cells, females, immune system and sex, anemia and immunity, anemia and infection

## Abstract

Mature erythrocytes are the major metabolic regulators by transporting oxygen throughout the body. However, their precursors and progenitors defined as CD71+ Erythroid Cells (CECs) exhibit a wide range of immunomodulatory properties. Here, we uncover pronounced sexual dimorphism in CECs. We found female but not male mice, both BALB/c and C57BL/6, and human females were enriched with CECs. CECs, mainly their progenitors defined as CD45+CECs expressed higher levels of reactive oxygen species (ROS), PDL-1, VISTA, Arginase II and Arginase I compared to their CD45− counterparts. Consequently, CECs by the depletion of L-arginine suppress T cell activation and proliferation. Expansion of CECs in anemic mice and also post-menstrual cycle in women can result in L-arginine depletion in different microenvironments *in vivo* (e.g. spleen) resulting in T cell suppression. As proof of concept, we found that anemic female mice and mice adoptively transferred with CECs from anemic mice became more susceptible to *Bordetella pertussis* infection. These observations highlight the role of sex and anemia-mediated immune suppression in females. Notably, enriched CD45+CECs may explain their higher immunosuppressive properties in female BALB/c mice. Finally, we observed significantly more splenic central macrophages in female mice, which can explain greater extramedullary erythropoiesis and subsequently abundance of CECs in the periphery. Thus, sex-specific differences frequency in the frequency of CECs might be imprinted by differential erythropoiesis niches and hormone-dependent manner.

## Introduction

The role of sex as an important biological variable has received significant attention in recent years. In 1993, the National Institutes of Health (NIH) decided to fill this gap by including women in clinical trials/studies to ensure that research outcomes apply to the whole population (NIH Revitalization Act of 1993 Public Law 103-43). With the emergence of personalized medicine and rejection of the one-size-fits-all therapeutic approaches, the significance of sex as a variable, and its impact on preventive, diagnostic, and therapeutic strategies have become more prominent (1). Males and females show explicit differences in their immunological profiles under physiological and pathological conditions. Generally, females mount a more robust cellular and humoral immune response, which results in efficient pathogen elimination and greater vaccine efficacy (2, 3). However, a strong immune response increases the risk of autoimmune diseases which is four times higher in females (3). In the context of innate immunity, there are several differences between males and females in terms of antigen recognition by Toll-like receptors (TLRs), frequency, and functionality of innate immune cells such as natural killer (NK) and dendritic cells (DCs) (4–6). Similarly, the impact of sex on different aspects of adaptive immunity has been widely documented. For example, it has been reported that human females have higher CD4+ T cells whereas CD8+ T cells are more abundant in males (3, 7). Although the Th1–Th2 dichotomy in males and females has been inconsistent (8), naïve CD4+ T cells in human females tend to produce more IFN-*γ* whereas their counterparts in males produce more IL-17 (9). Mouse studies on regulatory T cells (Tregs) have provided contradictory results but a human study has reported higher Tregs in males versus females (10). It is well acknowledged that genes associated with sex chromosomes, reproductive organs, and sex hormones are the main mechanisms behind such differences (2, 3, 6). Other environmental factors such as nutrition, microbiome, and lifestyle can affect immune responses in both sexes but these are usually considered as gender-associated factors (3).

Despite the extensive work on the influence of sex on different immune cell lineages, the effect of sex on erythrocytes has not been fully understood. Early studies have shown that men and women differ in their erythrocyte parameters including the size, count, and hemoglobin content (11). For example, reticulocytes were shown to be more enriched in the blood of women than men and suggested to be a compensatory response to the blood loss during menstruation (12). Moreover, higher levels of bone marrow-derived circulating progenitor cells with differential potentials for multiple lineages such as hematopoietic and endothelial cells in females are reported before the menopause age (13, 14). However, the impact of sex on the frequency of erythroid precursors and progenitors has not been well investigated.

In recent years, the physiological and pathological abundance of immunomodulatory erythroid precursors/progenitors defined as CD71+ Erythroid Cells (CECs) has received significant attention (15–17). CECs co-express CD71, the transferrin receptor, and TER119, the erythroid lineage marker, in mice but CD71 and CD235a in humans (18, 19). CECs mediate their immunosuppressive functions *via* cell–cell interaction and/or soluble mediators such as Arginase I (Arg I), Arginase II (Arg II) and reactive oxygen species (ROS) (15, 17, 19–22). CECs are highly abundant in neonatal mice up to 4-weeks of age regardless of sex and similarly up to 6 months in human newborns (18, 19, 23). Because of the extramedullary erythropoiesis (EE) (21), CECs expand in the peripheral blood during pregnancy in humans and mice (24, 25). EE induction requires hematopoietic stem cell (HSCs) activation and mobilization, which depends on the estrogen receptor-α (ERα) in HSCs (26). This suggests that sex hormones can influence EE that takes place mainly in the spleen and liver (21). Although adult mice and humans have a significantly lower frequency of CECs compared to neonates (18), there is no evidence about the potential impact of sex on their proportion. Therefore, we decided to investigate the frequency of CECs and their immunological properties in the spleen of female and male mice using two commonly used mouse strains, BALB/c and C57BL/6, and also in the peripheral blood of men and women.

## Material and Methods

### Animals

Male and female BALB/c and C57BL/6 mice were purchased from the Charles River Institute. All animals were maintained and bred under pathogen-free conditions within the animal care facility at the University of Alberta. Throughout our studies, we used age-matched male and female mice (8–10 weeks). For anemia induction, BALB/c mice were injected intraperitoneally (i.p.) with 60 µg of the anti-TER119 antibody (Bio XCell).

### Ethics Statement

This study was conducted under the recommendations in the Guide for the Care and Use of Laboratory Animals of the Canadian Council for Animal Care. The protocol was approved by the Animal Ethics Board of the University of Alberta (Protocol # AUP00001021). Similarly, the appropriate Institutional Review Boards at the University of Alberta approved the human studies (Protocol # Pro00046080). All study participants gave written informed consent to participate in the study.

### Human Sample Collection and Processing

Blood samples were obtained from both male and female age-matched healthy controls. The study subjects were between the age of 20 and 50 without any underlying conditions and no clinical evidence of anemia. For some studies, blood samples were collected from females a week before their menstrual and 3–5 days’ post-menstrual cycle. Thereafter, peripheral blood mononuclear cells (PBMCs) were isolated over Ficoll–Hypaque gradients. For CEC isolation, blood samples were stained using anti-CD71 or isotype control biotin-conjugated antibody and fractioned using streptavidin-linked magnetic beads (Miltenyi Biotec) (19).

### Antibodies and Flow Cytometry

Fluorophore or biotin-conjugated antibodies with specificity to mouse cell surface antigens and cytokines were purchased from the BD Biosciences or Thermo Fisher Scientific. Specifically, the following antibodies were used for mice: anti-CD71 (R17217 and C2F2), anti-Ter119 (TER-119), anti-CD45 (30-F11), anti-VISTA (MIH64), anti-PDL-1 (MIH5), anti-CD11b (M1/70), anti-CD11c (N418), anti-CD3 (145-2C11), anti-CD4 (GK1.5), anti-CD8a (53-6.7), anti-IFN-*γ* (XMG1.2), anti-F4/80 (6F12), anti-CD169 (Siglec-1, 3D6.112), anti-VCAM-1 (51-10C9), anti-Arg I (IC5868A R&D) and anti-Arg II (ab81505, Abcam). For human studies, the following fluorophore or biotin-conjugated antibodies with specificity to surface markers or cytokines were used: anti-CD3 (HIT3a), anti-CD4 (RPA-T4), anti-CD8 (RPA-T8), anti-CD45 (H-130 or 2D1), anti-IFN-*γ* (4SB3), anti-CD71 (MA712), Ki67 (20Raj1), anti-CD69 (FN50), anti-Arg II (ab81505, Abcam), and anti-CD235A (HIR2). ROS staining (Sigma) was performed by flow cytometry per the manufacturer’s protocols and our previous reports (25, 27). Live/dead fixable dead cell staining (ThermoFisher) was used to exclude dead cells in flow cytometry. For proliferation studies, CFSE labeling was performed per our previous protocols (28, 29). Paraformaldehyde fixed cells were acquired by flow cytometry using the LSRFORTESSA flow cytometer (BD) and analyzed with FlowJo software (version 10.7.1).

### Co-Culture and Stimulation

For *in vitro* intracellular cytokine staining, human PBMCs or mice splenocytes were cultured and stimulated with anti-CD3/CD28 (3 and 1 µg/ml, respectively) in RPMI media supplemented with 10% FBS for 6 h in the presence or absence of CECs according to our previous reports (30, 31). For co-culture, a fixed number (1 × 10^5^) of isolated T cells were seeded onto 96-well round bottom plates individually or with isolated CECs at different ratios with Brefeldin A (10 μg/ml). For some experiments, L-arginine (2 mM) was added at the time of stimulation to abrogate the effects of Arg II *in vitro* (19). For mice studies, splenocytes were harvested, and single-cell suspension was made by grinding between sterile frosted glass slides in RBC lysis buffer and filtering through nylon mesh as we have reported elsewhere (19). Splenocytes were washed by centrifugation and used for subsequent *in vitro* studies. In some experiments, T cells were isolated using the T cells isolation kit (Stem Cell Technologies) and CECs using a biotin-conjugated antibody and fractioned using streptavidin-linked magnetic beads (Miltenyi Biotec). To distinguish donor cells in co-culture experiments, we used mice with different congenic markers (e.g. CD45.2 and CD45.1 in the case of C57BL/6) of labeled donor cells with the CFSE dye (BALB/c mice).

### Infection Model

*Bordetella pertussis* strain Tomaha I was cultured on Bordet–Gengou agar supplemented with 15% sheep blood as we described elsewhere (32, 33). Adult female or male (8 weeks) anemic or non-anemic mice were anesthetized by inhalation of isoflurane and administered intranasally with 50 µl of 5 × 10^6^ colony-forming units (CFUs) of *B. pertussis* (34).

### Adoptive Cell Transfer and Purification

Splenocytes from anemic mice were processed into single cell suspensions by grinding between sterile frosted glass slides in RBC lysis buffer and filtering through nylon mesh as we have reported elsewhere (19). As described above, CECs were purified by negative selection using biotin-conjugated antibodies and streptavidin linked magnetic beads (Miltenyi Biotec) according to our previous reports (19). Isolated CECs from anemic mice (1 × 10^7^) first stained with the CFSE-dye then injected intravenously into the tail vein of recipient mice 24 h prior infection. Control mice were administered through the tail vein with 1 × 10^7^ mature red blood cells. The single cell suspension of splenocytes was processed in the absence of RBC lysis buffer then mature RBCs and CECs were isolated by positive selection using biotin-conjugated anti-TER119 antibody (Thermo Fisher Scientific). In the next step, we used the anti-CD71 antibody on PE followed by anti-PE beads (Miltenyi Biotec) to exclude CECs.

### Statistical Analysis

Statistical analyses were carried out using the GraphPad Prism (version 8) software using the appropriate statistical tests for various data sets. All statistically significant values were identified as having a p-value rate of <0.05.

## Results

### Female Mice Have a Higher Proportion of CECs in Their Spleens

**Figure 1:**
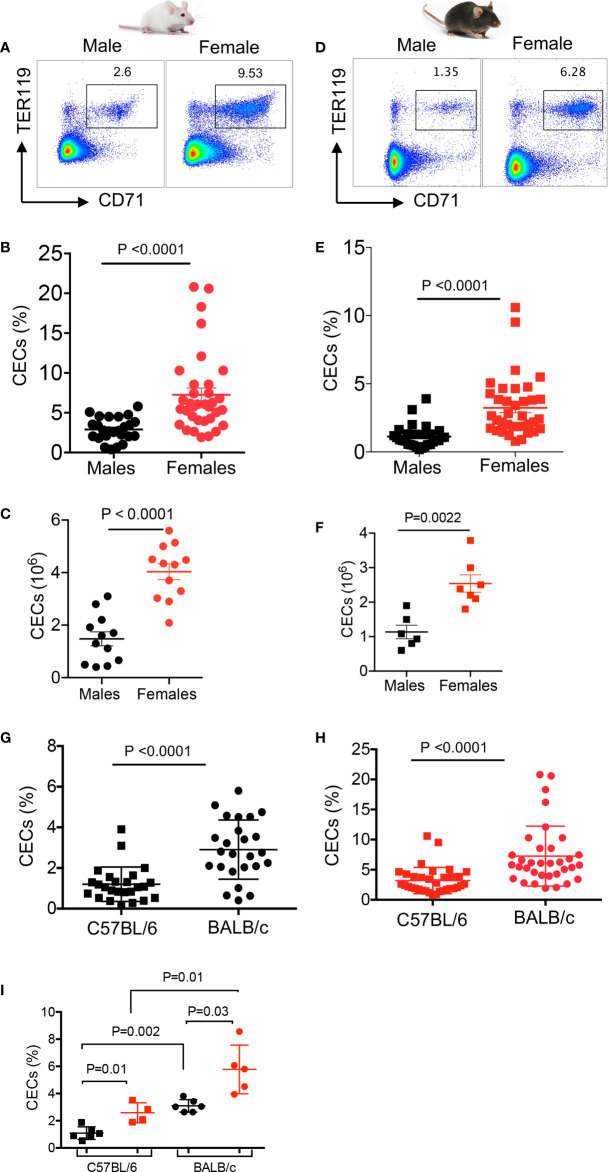
Higher frequency of CECs in female versus male mice. **(A)** Representative flow cytometry plots, **(B)** cumulative data of percentages, and **(C)** absolute numbers of CECs in spleens of BALB/c male and female mice. **(D)** Representative flow cytometry plots, **(E)** cumulative data of percentages, and **(F)** absolute numbers of CECs in spleens of C57BL/6 male and female mice. **(G)** Cumulative data comparing percentages of CECs in spleens of C57BL/6 and BALB/c male mice. **(H)** Cumulative data comparing percentages of CECs in spleens of C57BL/6 and BALB/c female mice. **(I)** Cumulative data comparing percentages of CECs in spleens of C57BL/6 male and female, and BALB/c male and female littermates at 8 weeks old. Each point represents data from an individual mouse, representative of at least three independent experiments. Bar, mean ± standard error.

We have previously reported that CECs expand during pregnancy and are important in feto-maternal tolerance in mice (24). Therefore, we decided to quantify the frequency of CECs in the spleen of adult female and male mice by examining two commonly used mice strains; BALB/c and C57BL/6. We found significantly higher percentages and absolute numbers of CECs in the spleen of adult females compared to male BALB/c mice (**Figures 1A–C**, and **Supplemental Figure S1A**). Although BALB/c males had significantly higher body weight (**Supplemental Figure S1B**), their spleen weights were similar to females (**Supplemental Figure S1C**). Similarly, we observed significantly higher percentages and absolute numbers of CECs in the spleen of adult C57BL/6 female mice (**Figures 1D–F**). We also noted that C57BL/6 male mice had significantly higher body weight but similar spleen weight compared to female mice (**Supplemental Figures S1D, E**). Interestingly, the frequency of CECs was significantly higher in the spleen of BALB/c mice, both sexes, compared to their C57BL/6 counterparts (**Figures 1G, H** **)**. Furthermore, to prevent potential confounding factors such as variation between litters, we decided to quantify the frequency of CECs in littermates when reaching adulthood (8 weeks). Once again, we found a significant difference in the frequency of CECs between male and female mice regardless of the mouse strain (**Figure 1I**). Overall, these observations confirmed a higher frequency of splenic CECs in females compared to males in both strains of mice.

### Splenic CECs in Adult Mice Express ROS, Arg II, and Arg I

**Figure 2:**
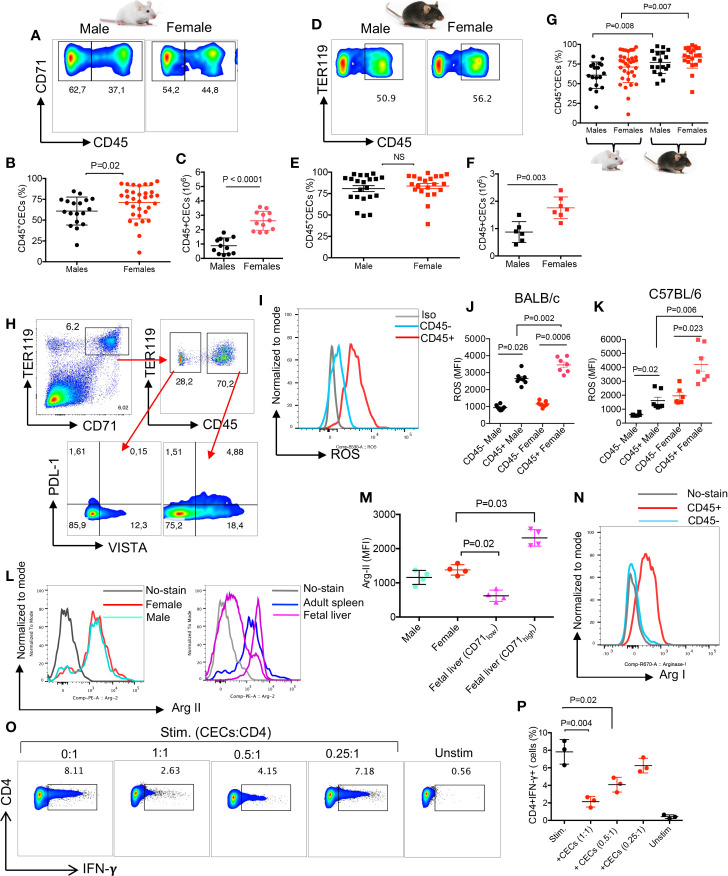
CECs in adult mice express Arg II and ROS and suppress IFN-*γ* production by T cell. **(A)** Representative flow cytometry plots, **(B)** cumulative data of percentages, and **(C)** absolute numbers of CD45+/CD45-CECs in the spleen of BALB/c male and female mice. **(D)** Representative flow cytometry plots, **(E)** cumulative data of percentages, and **(F)** absolute numbers of CD45+/CD45−CECs in the spleen of C57BL/6 male and female mice. **(G)** Cumulative data comparing percentages of CD45+CECs in spleens of BALB/c and C57BL/6 male and female mice. **(H)** Representative flow cytometry plot of PDL-1 and VISTA expression in CD45− and CD45+ CECs from a female BALB/c mouse. **(I)** Histogram plots, and **(J)** cumulative data of the mean fluorescence intensity (MFI) for ROS expression in CD45− and CD45+CECs in male and female BALB/c mice. **(K)** Cumulative data of MFI for ROS expression in CD45+ BALB/c male and female C57BL/6 mice. **(L)** Histogram plots, and **(M)** cumulative data of Arg II expression (MFI) in total CECs in male versus female mice compared to fetal liver CD71^low^ and CD71^high^ CECs. **(N)** Representative histogram plot of Arg I expression in splenic CD45+ and CD45−CECs of a female BALB/c mouse. **(O)** Representative flow plots, and **(P)** cumulative data of IFN-***γ*** production by CD4+ T cells in the absence or presence of CECs at different ratios (CECs : CD4) after stimulation with anti-CD3/CD28 antibodies for 6 h. Unstimulated (Unstim), stimulated with anti-CD3/CD28 (stim). Each point represents data from an individual mouse, representative of at least three independent experiments. Bar, mean ± standard error.

The immunosuppressive properties of CECs *via* Arg II expression has been widely documented in neonatal mice (18, 19, 24). Therefore, we decided to further characterize the functionality of CECs in adult mice. We found a significantly higher percentages and absolute numbers of splenic CD45+CECs in female BALB/c mice compared to their male counterparts (**Figures 2A–C**). While in terms of percentages of CD45+CECs we did not find any significant difference between male and female C57BL/6 mice (**Figures 2D, E** **)**, females had significantly higher numbers of CECs in their spleens when total cell count was evaluated (**Figure 2F**). However, we noticed that the frequency of CD45+CECs was significantly higher in both C57BL/6 male and female mice compared to their BALB/c counterparts (**Figure 2G**). CECs are a heterogeneous population of erythroid progenitors and precursors (21, 23). Erythroid progenitors downregulate CD45 as they mature; therefore, CD45+CECs reflect erythroid progenitors (35). These observations suggest that C57BL/6 mice possess more erythroid progenitors in their spleens. CECs are more abundant in the spleen of neonatal mice (19), however, the proportion of CD45+CECs appears to be much higher in adults compared to neonatal mice (**Supplemental Figure S1F**, and **Figure 2G**).

CECs express different co-inhibitory molecules such as PDL-1 and the V-domain Ig suppressor of T cell activation (VISTA) as we have reported elsewhere (24, 36). Therefore, we decided to measure the surface expression of PDL-1 and VISTA in CD45+ versus CD45−CECs. We found that CD45+CECs were the dominant cells expressing PDL-1 and VISTA compared to their CD45−CEC siblings (**Figure 2H**). However, there was no significant difference in the percentages of VISTA+ and PDL-1+ CECs between males and females in both mice strains (**Supplemental Figures S1G–J**). It is worth to mentioning that CECs from male C57BL/6 but not female mice appeared to have significantly a greater proportion of PDL-1+CECs compared to their counterparts in BALB/c mice (**Supplemental Figure S1K**). However, there was only a significant difference in the frequency of VISTA+CECs in female C57BL/6 compared to female BALB/c mice (**Supplemental Figure S1L**). The surface expression of PDL-1 and VISTA on adult CECs suggests that these cells similar to their counterparts in neonatal and pregnant mice may exert their biological functions *via* cell–cell interactions (e.g. PD-1:PDL-1) (15, 24). The higher expression of PDL-1 and VISTA in CECs of C57BL/6 versus BALB/c mice could be explained by the increased proportion of CD45+CECs in these mice compared to BALB/c mice (**Figure 2G**). Next, we measured the expression of ROS in CD45+ and CD45−CECs, which showed significantly higher ROS expression in CD45+CECs compared to their negative counterparts regardless of the mouse strain (**Figures 2I–K**), as we have reported in newborns (23). Also, we noted significantly higher ROS expression in CD45+CECs in both BALB/c and C57BL/6 female mice compared to their male counterparts (**Figures 2I–K** and **Supplementary Figure S2A**). However, we did not find any significant difference in the expression of ROS by CD45+CECs between C57BL/6 versus BALB/c female mice (**Supplemental Figure S2B**). Next, we decided to quantify the expression of Arg I/II as the most potent regulators of T cell proliferation and function (37, 38). In agreement with neonatal CECs (18, 19), we found a similar expression level of Arg II in splenic CECs of both female and male mice regardless of the mouse strain (**Figures 2L, M** **)**. Since CECs in the fetal liver of mice express high levels of Arg II (24), we compared Arg II expression in splenic CECs of adult mice versus fetal liver. We found that although the CD71^low^ subpopulation of fetal liver CECs expressed significantly lower levels of Arg II, the CD71^high^ subpopulation expressed significantly higher levels of Arg II compared to their counterparts in the spleen of adult mice (**Figures 2L, M** and **Supplemental Figure S2C**). This is related to the abundance of CD45 expressing cells in the CD71^high^ subpopulation (23). We also measured the expression of Arg I in CECs and found significantly higher expression of Arg I in CD45+CECs versus their negative counterparts in both sexes/mice strains (**Figure 2N** and **Supplementary Figures S2D, E**). Moreover, we observed that CD45+CECs in BALB/c female mice had significantly higher expression of Arg I compared to their counterparts in male mice (**Supplementary Figure S2D**). However, this was not the case for CD45+CECs in C57BL/6 mice (**Supplementary Figure S2E**). Finally, we noted significantly higher Arg I expression in CD45+CECs in female BALB/c compared to female C57BL/6 mice (**Supplementary Figure S2F**). These results suggest that CECs in adult mice mediate-immunosuppression possibly *via* cell–cell interactions and/or soluble factors such as ROS and Arg I/II.

### Splenic CECs in Adult Female Mice Suppress T Cell Cytokine Production and Proliferation

**Figure 3:**
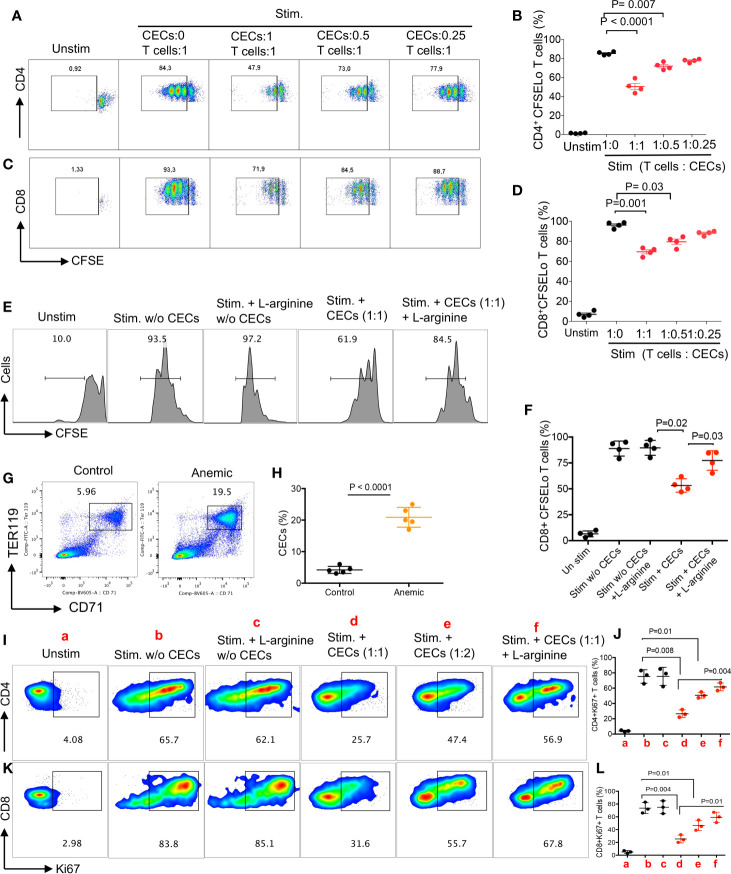
CECs from female mice suppress T cell activation and proliferation. **(A)** Representative flow cytometry plots, and **(B)** cumulative data of CD4+ T cell proliferation as measured by CFSE dilution in the absence or presence of CECs at indicated ratios following stimulation with anti-CD3/CD28 for 3 days. **(C)** Representative flow cytometry plots, and **(D)** cumulative data of CD8+ T cell proliferation in the absence or presence of CECs at indicated ratios following stimulation with anti-CD3/CD28 for 3 days. **(E)** Representative flow cytometry plots, and **(F)** cumulative data of CD8+ T cell proliferation in the absence or presence of CECs at indicated ratios following stimulation with anti-CD3/CD28 for 3 days with or without (w/o) L-arginine supplementation (2 mM). **(G)** Representative flow cytometry plots, and **(H)** cumulative data of CECs in untreated (control) versus treated female mice with the anti-TER-119 antibody. **(I)** Representative flow cytometry plots, and **(J)** cumulative data for Ki67 expression in CD4+ T cells following stimulation for 24 h with anti-CD3/CD28, in the absence or presence of CECs (different indicated ratios) and with or without (w/o) supplementation with L-arginine (2 mM). **(K)** Representative flow cytometry plots, and **(L)** cumulative data for Ki67 expression in CD8+ T cells following stimulation for 24 h with anti-CD3/CD28, in the absence or presence of CECs (different indicated ratios) and with or without (w/o) supplementation with L-arginine (2 mM). Each point represents data from an individual mouse, representative of at least three independent experiments. Bar, mean ± standard error.

As proof of concept, we isolated T cells and CECs, then performed intracellular cytokine staining for IFN-*γ* following stimulation with anti-CD3/CD28 antibodies in the presence or absence of CECs for 6 h. We found that CECs from BALB/c reduced IFN-*γ* expression by CD4+ T cells in a dose-dependent manner (**Figures 2O, P** **)**. Next, we labeled T cells with CFSE, stimulated them with anti-CD3/CD28 antibodies in the absence or presence of CECs with purity >95% (**Supplemental Figure S2G**) for 3 days. We found that CECs suppressed both CD4+ and CD8+ T cell proliferation *in vitro* in a dose-dependent manner (**Figures 3A–D**). Since CD45+CECs were significantly higher in female BALB/c mice compared to their male counterparts (**Figures 2A, B**), and CD45+CECs had higher expression of PDL-1, VISTA, Arg I and ROS (**Figures 2H–K, N**), we reasoned to compare their immunosuppressive properties in female versus male BALB/c mice. These observations indicated a greater immunosuppression of T cell proliferation by CECs from female compared to their male counterparts (**Supplemental Figures S3A, B**). To better understand the mechanism underlying T cell suppression by adult CECs, we conducted similar proliferation assays in the presence and absence of CECs supplemented with or without L-arginine (2 mM). We observed that L-arginine supplementation partially but significantly reversed the immunosuppressive properties of CECs *in vitro* (**Figures 3E, F** **)**. Although our data do not exclude the possibility that CECs *via* ROS or cell–cell interactions may also exhibit immunosuppressive properties, we believe Arg I/II expression is one mechanism of CECs-mediated T cell suppression in adult mice.

### Anemia Promotes the Expansion of CECs in Female and Male Mice

As anemia is more common in women than men and we have observed a reverse correlation between the hemoglobin levels with percentages of CECs in COVID-19 patients, we sought to investigate whether anemia-induced CECs exhibit immunosuppressive properties. Adult female and male mice were injected i.p. with the anti-TER119 antibody and 5 days later evaluated for the frequency of splenic CECs. As shown in **Figures 3G, H**, the anti-TER119 antibody administration resulted in a significant expansion of splenic CECs in female and male mice (**Supplemental Figures S3C, D**). Similar to our previous report (19), we found that anemia-induced CECs did not suppress TNF-α production by CD11b+ cells *in vitro*. However, these isolated CECs from anemic mice suppressed the proliferation of both CD4+ and CD8+ T cells as measured by Ki67 (39) when stimulated with anti-CD3/CD28 antibodies *in vitro* (**Figures 3I–L**). Notably, the inhibitory property of CECs from anemic mice on T cell proliferation was mainly Arg-dependent (**Figures 3I–L**). These observations show the differential capacity of anemia-induced CECs compared to the neonatal CECs (19) as neonatal CECs have lower proportion of CD45+CECs (**Supplemental Figure S1F**). Subsequently, we observed higher Arg II expression in anemia-induced CECs compared to their mature red blood cell counterparts (**Supplemental Figure S3E**). Although we did not find any significant difference in the percentages of CD4+ and CD8+ T cells in the spleen of male versus female mice (**Supplemental Figure S3F**), higher frequency/absolute number of CECs in females may predispose their T cells to a more pronounced CECs-mediated immunosuppression. Overall, these observations provide a novel insight into the impact of anemia-induced CECs on T cell function.

### Anemia and Anemia-Induced CECs Enhance Susceptibility to *Bordetella pertussis* Infection

**Figure 4:**
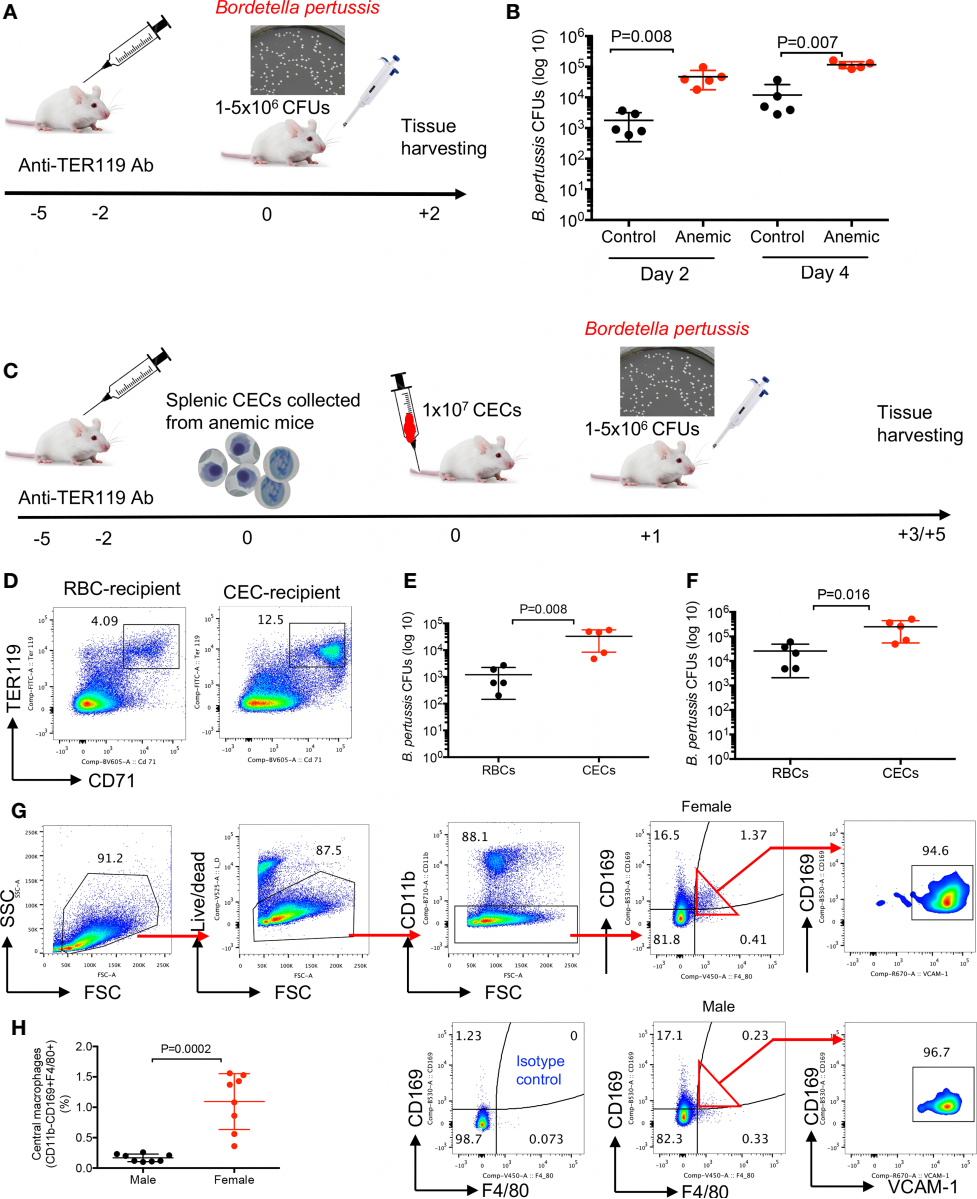
Anemia and anemic-induced CECs enhance susceptibility to infection in female mice. **(A)** A schematic representation of the anti-TER119 antibody treatment and intranasal infection of mice with *B. pertussis* bacteria. **(B)** Cumulative data of number of isolated *B. pertussis* shown as colony forming units (CFUs) from the lung homogenates of control (IgG2b-treated) and anemic (anti-TER119-treated) mice 2 and 4 days’ post-infection. **(C)** A schematic representation of the anti-TER119 antibody treatment, isolation of splenic CECs, tail vein injection of CECs and intranasal infection of mice with *B. pertussis*. **(D)** Representative flow cytometry plots of percentages of CECs in the spleen of control versus CEC-treated female mouse. **(E)** Cumulative data of number of isolated *B. pertussis* from the lung homogenates of control (RBC-injected) and CEC-injected mice 2, and **(F)** 4 days’ post-infection. **(G)** Representative flow cytometry plots, and **(H)** cumulative data of central macrophages in female versus male mice. Each point represents data from an individual mouse, representative of at least two independent experiments. Bar, mean ± standard error.

First, we evaluated the influence of anemia on infection susceptibility in female and male mice. As shown in **Figure 4A**, BALB/c female mice were injected (i.p.) with the anti-TER119 antibody (60 µg/injection) at 5 and 2 days before the infection, and control mice received the isotype control antibody (IgG2b). Mice were intranasally challenged with *B. pertussis* (1–5 × 10^6^ CFUs) (40). Two and four days later the bacterial load was quantified by the serial culture dilution of lung homogenates on the Bordet–Gengou (BG) agar media (41, 42). We found a significant increase in the bacterial load in the lungs of anemic female mice compared to the control group (**Figure 4B**). We performed similar studies on anemic male mice but the outcome was different. While anemic male mice exhibited significantly higher susceptibility to *B. pertussis* infection at day 2 post-infection, the bacterial load in the lungs of anemia male mice was not significantly different compared to controls at day 4 post-challenge (**Supplemental Figure S3G**). To avoid other potential effects of anemia in treated animals and to demonstrate a direct connection between the anemia-induced CECs and infection susceptibility, we decided to perform adoptive transfer of CECs. Splenic CECs from the anemic BALB/c female mice (8 weeks) were enriched and transferred (1 × 10^7^ cells) *via* the tail vein injection into another group of female BALB/c mice (**Figure 4C**). In general, we use two to four spleens from healthy female mice for isolating 1 × 10^7^ CECs. However, because of the expansion of CECs in anemic mice, we require one to two mice to obtain 1 × 10^7^ CECs. Control mice received mature red blood cells. The presence of CFSE-labeled adoptively transferred CECs was confirmed in the spleen of recipient mice the next day by flow cytometry (**Figure 4D**) before the intranasal challenge with *B. pertussis* (5 × 10^6^ CFUs). Two days later the bacterial load was quantified by the serial culture dilution of lung homogenates on the BG agar media, and we found enhanced susceptibility of CEC-recipient mice to *B. pertussis* infection (**Figure 4E**). This increased bacterial load remained significantly higher even during 4-day post-infection (**Figure 4F**). Thus, the enhanced susceptibility of anemic mice to *B. pertussis* infection supports the immunosuppressive property of CECs *in vivo*.

### Higher Abundance of Central Macrophages in the Spleen of Female Mice

To better understand the mechanism underlying significantly greater percentages of CECs in the spleen of female versus male mice, we reasoned to determine EE in female versus male mice. To test this hypothesis, we studied the presence of erythroblastic islands (EBIs) (43) in the spleen of female and male adult mice. These islands are niches where central macrophages interact closely with RBCs in their different stages of proliferation/maturation and engulf free nuclei as they are extruded from the reticulocytes (44). We analyzed the presence of central macrophages defined as CD11b^−^CD169^+^F4/80^+^VCAM1^+^ according to a recent study (45). It is worth mentioning that under rapid stress erythropoiesis after treatment with phenylhydrazin (PHZ) the phenotype of splenic central macrophages appears to be different. During the recovery process from anemia early EBI niches are enriched with phenotypically more monocyte-like cells expressing high CD11b and Ly6C but low F4/80, CD169and Vcam-1 levels (46). As shown in **Figures 4G, H** and **Supplemental Figures S3H, I**, we observed significantly higher proportion and absolute number of central macrophages in spleens of female compared to male mice either BALB/c or C57BL/6 strain. Therefore, enriched central macrophages in the spleen of female mice might in part explain the abundance of EBIs in females.

### Expanded CECs in the Peripheral Blood of Human Females Suppress T Cell Proliferation

**Figure 5:**
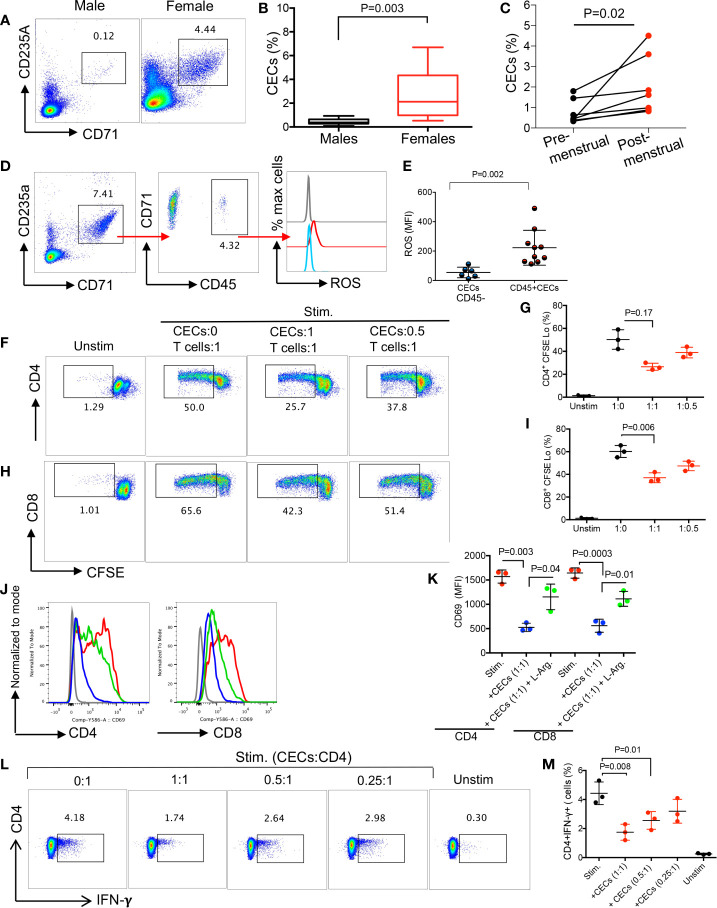
CECs are more abundant in human females and suppress T cell activation and IFN-*γ* production. **(A)** Representative flow cytometry plots, and **(B)** cumulative data of percentages of CECs in males versus females (>15 subjects/group). **(C)** Cumulative data of % CECs in females pre-and post-menstrual cycle. **(D)** Representative flow cytometry plots of percent CD45+ subpopulation and ROS expression in CECs. **(E)** Cumulative data of MFI for ROS expression in CD45− and CD45+ CECs in women. **(F)** Representative flow cytometry plots, and **(G)** cumulative data of CD4+ T cell proliferation as measured by CFSE dilution in the absence or presence of CECs at indicated ratios following stimulation with anti-CD3/CD28 for 3 days. **(H)** Representative flow cytometry plots, and **(I)** cumulative data of CD8+ T cell proliferation in the absence or presence of CECs at indicated ratios following stimulation with anti-CD3/CD28 for 3 days. **(J)** Histogram plots, and **(K)** cumulative data of CD69 expression on CD4+ and CD8+ T cells in the absence or presence (1:1 ratio) of CECs following stimulation with anti-CD3/CD28 for 24 h with or without supplementation with L-arginine (2 mM) in females. **(L)** Representative flow plots, and **(M)** cumulative data of IFN-***γ*** production by CD4+ T cells in the absence or presence of CECs at different ratios (CECs : CD4) after stimulation with anti-CD3/CD28 for 6 h. Unstimulated (Unstim), stimulated with anti-CD3/CD28 (stim). Each point represents data from an individual woman, data are from three different study subjects. Bar, mean ± standard error.

We have previously reported a physiologically enriched proportion of CECs in the late stage of pregnancy in pregnant women (25). In this study, we compared the frequency of CECs in the peripheral blood mononuclear cells (PBMCs) of adult non-pregnant women compared to their age-matched males. We found females had significantly higher levels of CECs in their PBMCs compared to males (**Figures 5A–B**, and **Supplementary Figure S3J**). More interestingly, we noted a significant expansion of CECs in PBMCs of women post-menstrual cycle (**Figure 5C**).

Similar to mouse CECs, we observed that CD45+CECs were the most dominant source of ROS expression. However, CD45+CECs constituted a small proportion of CECs in human females (**Figures 5D, E** **)**. Next, we decided to evaluate their immunosuppressive properties by performing the CFSE proliferation assay. Similar to our observations in mice, CECs from human females exhibited immunosuppressive properties in a dose-dependent manner when co-cultured with isolated T cells and stimulated with anti-CD3/CD28 antibodies for 3 days (**Figures 5F–I**). To better understand the mechanism associated with their immunosuppressive properties, we were unable to measure the expression of Arg II since human CECs get lysed once exposed to the permeabilization buffer during Arg II intracellular staining as reported elsewhere (25). However, we have previously detected the Arg II gene in human CECs in pregnant women by qPCR (25). Notably, we were able to detect Arg II in COVID-19 patients since SARS-CoV-2 infection makes CECs resistant to the permeabilization buffer (22). Therefore, we decided to investigate whether CECs in human females *via* Arg II mediate immunosuppression *in vitro*. Isolated T cells were co-cultured with the isolated CECs with purity >95% (**Supplemental Figure S3K**) at a 1:1 ratio in the absence or presence of L-arginine (2 mM) following stimulation with anti-CD3/CD28 antibodies overnight. We observed that CECs suppressed both CD4+ and CD8+ T cell activation measured by CD69; however, L-arginine supplementation partially reversed this inhibitory effect (**Figures 5J, K** **)**. Finally, we measured IFN-*γ* secretion by T cells following stimulation with anti-CD3/CD28 antibodies in the presence or absence of CECs. We found that CECs in a dose-dependent manner suppressed IFN-*γ* production by CD4+ T cells as measured by the intracellular cytokine staining (**Figures 5L, M** **)** and similarly in by CD8+ T cells (data not shown). These observations suggest that the abundance of CECs in human females can suppress T cell activation/proliferation.

## Discussion

Sex as a biological variable affects immune responses to self and non-self-antigens, and it influences multiple aspects of innate and adaptive immunity (3, 47). However, the effect of sex on CECs has not been well appreciated in the past. In this study, we have provided a novel insight into the influence of sex on erythropoiesis as demonstrated by the differential frequency of CECs in males versus females. We show that suppression of T cells is a common property of both murine and human CECs that are enriched in the female. However, this was more pronounced in female than male BALB/c mice possibly because of a higher proportion of CD45+CECs (erythroid progenitors) in females. CECs, particularly, CD45+CECs suppress T cell proliferation and activation *via* Arg I/II and ROS. Recent advancement in the field has provided more depth into the role of CECs in different physiological and pathological conditions. Immunosuppressive properties of physiologically enriched CECs have been widely described in the neonatal period (19, 20). Neonatal CECs *via* the expression of Arg II can suppress activation of myeloid-derived cells and impair humoral and cellular immune responses against infection in mice (18, 19, 30). In addition to Arg II, CD45+CECs in both neonatal mice and human newborns can suppress cytokine production by myeloid cells *via* ROS production and Arg I (23). Unlike Tregs that require days to exert their immunosuppressive functions (48), CECs exhibit their regulatory function (e.g. inhibition of cytokine production) in a matter of hours. Moreover, CECs expand during pregnancy in both humans and mice and play an important role in feto-maternal tolerance (24, 25, 49) as their depletion results in fetal resorption in an allogenic mouse model (24). In this study, we observed CECs were more abundant in females and they expand during the post-menstrual cycle. The increased frequency of CECs post-menstrual cycle and in anemic mice suggest that iron deficiency or blood loss in females might be a contributing factor for the expansion of CECs. It is well-documented that men and women have similar erythropoietin levels but women have lower hemoglobin levels (50). Although we were unable to measure hemoglobin levels in our study subjects, we have observed a negative correlation between the hemoglobin level and the frequency of CECs in COVID-19 patients (22). This observation may support the concept of anemia as a driving factor in the expansion of CECs in the periphery. However, further studies in larger cohorts are required to examine this hypothesis. In general, anemia disproportionally affects women (51) and the expansion of CECs might serve as a compensatory mechanism for anemia. Previous studies suggested that acute anemia causes tissue hypoxia, which enhances the production of erythropoietin (Epo). Subsequently, Epo mobilizes cells with erythroid lineage progenitors from the bone marrow to the spleen where they expand and mature (52). However, this idea has been challenged by the discovery of resident erythroid progenitors in the spleen of mice which upon stress erythropoiesis activate and expand (53). This may resemble resident stem cells in mammalian intestinal epithelium involved in homeostasis and the epithelium regeneration (54). The generation of erythrocytes outside of medullary spaces of the bone marrow defined as EE occurs at a very small rate in the spleen of adult mice (55). Under normal circumstances, erythrocytes following maturation enter the blood circulation (15, 21) while under pathological conditions EE is considered as the main cause for the abundance of erythroid precursors in the periphery (21). This may occur as a result of passive incontinence of hematopoietic cells release from the site of EE (56). Also, sex hormones may contribute to the higher CECs in females. For example, ERα is highly expressed on HSCs, and 17β-estradiol, an estrogen agonist of ERα, enhances splenic HSC proliferation and subsequently promotes EE in females in particular during pregnancy (57). In agreement, we found significantly more frequency of splenic central macrophages in female than in male mice. This suggests that female mice might have more splenic EBIs, however, further studies are required to prove this hypothesis. Although performing such comparison in human subjects was not feasible, there is a possibility to speculate a similar pattern in the spleen of human females compared to males. Nevertheless, this concept merits further investigations.

The EE has been reported in chronic conditions such as HIV and cancer (17, 27), which results in the expansion of CECs in the periphery. Even though the exact role of expanded CECs in females and in the context of anemia has not been well studied, we believe CECs may impair innate and adaptive immune responses against infections and cancer (17, 19, 30). However, this can be controversial since females represent 80% of all of the autoimmune diseases (58). Moreover, in the experimental autoimmune encephalomyelitis (EAE) model and PBMCs from multiple sclerosis patients, females exhibit greater Th1 responses, and IFN-*γ* production compared to males (9). However, based on our observations the expansion of CECs might be beneficial in certain circumstances by preventing hyper-immune activation (25). Notably, anemia and iron-deficiency have been associated with the expansion of Tregs with a protective role in the EAE model (59). Although the authors in this study did not investigate the presence of CECs, it’s likely that anemia-induced CECs may contribute to the induction of Tregs (36). It is worth mentioning that CECs-mediated impaired production of IFN-*γ* as a potent inhibitor of erythropoiesis (60) may support the maintenance of erythropoiesis.

Moreover, the abundance of CECs in anemic individuals may in part explain the underlying mechanism of impaired immunity in anemia (61, 62). This concept was supported by the enhanced susceptibility of anemic mice to *B. pertussis* infection. Notably, the increased bacterial load in the lungs of CEC recipient mice demonstrate a direct connection between the anemia-induced CECs and susceptibility to infection. Although anemia-induced CECs did not suppress TNF-α production by myeloid cells *in vitro*, they may suppress innate immune response against *B. pertussis in vivo*. This hypothesis is supported by our previous observations that the depletion of CECs enhanced the recruitment of NK cells and antigen presenting cells (APCs) into the lungs of mice (18). Subsequently, this resulted in the elevation of protective cytokines (e.g. IFN-*γ*, TNF-α and IL-12) and lower *B. pertussis* in the lungs of mice (18). However, it is possible to suggest that this effect might be more pronounced and persistent in the context of chronic anemia and continuous expansion of CECs as we have shown in anemic mice. Of note, the consistency of the excessive *B. pertussis* infection rate in females is well documented (63). In agreement, we found female anemic more exhibited higher susceptibility to *B. pertussis* than their male counterparts. This explains the influence of sex in this respiratory infection, however, the role of CECs in this context merits further investigation. Taken together, our novel findings highlight the differential influence of sex on CECs and reveal that CECs should be considered as a sex-associated variable in both humans and mice. Considering the immunomodulatory properties of CECs, our observations could have implications in animal experimental design and data interpretation. However, we are aware of multiple study limitations such as the small number of human females for pre-post-menstrual cycle studies that due to the pandemic we were restricted in recruiting more study subjects. Also, we were unable to correlate the frequency of CECs with the hemoglobin levels in healthy individuals although we have conducted such studies in COVID-19 patients (22). Another limitation of our study was related to using total CD4+ T cells instead of excluding Tregs when we performed co-culture studies with CECs. Further studies are required to determine whether exclusion of Tregs results in a different outcome.

Our data indicate that CECs in both male and female mice exhibit immunosuppressive properties; however, it was more pronounced in female mice. Because of the extremely low frequency of CECs in human males it was impossible to isolate enough CECs for performing functional studies.

## Data Availability Statement

The original contributions presented in the study are included in the article/**Supplementary Material**. Further inquiries can be directed to the corresponding author.

## Ethics Statement

The studies involving human participants were reviewed and approved by The Ethics Board at the University of Alberta. The patients/participants provided their written informed consent to participate in this study. The animal study was reviewed and approved by The Ethics Board at the University of Alberta.

## Author Contributions

SM performed most of animal studies and pre-post menstrual studies, analyzed the data, and wrote part of the introduction. PK performed a wide range of animal studies, including functional assays, comparing the frequency of CECs in male and female mice, and analyzed the data. MH performed some of animal studies for the frequency of CECs and central macrophages. IO performed some of the human related studies. SS assisted in blood collection and processing from human subjects. SE conceived the original idea, designed, and supervised all the research, secured resources, performed some of the functional assays, assisted in data analysis, and wrote the manuscript. All authors contributed to the article and approved the submitted version.

## Funding

This study was supported by the Canadian Institute for Health Research (CIHR) through a Foundation Grant and a New Investigator Award (both to SE). Also, this study was supported by an Innovation Grant from the Women and Children’s Health Research Institute. Nevertheless, the funding bodies had no role in the design of the study, data collection, analysis, and interpretation of data.

## Conflict of Interest

The authors declare that the research was conducted in the absence of any commercial or financial relationships that could be construed as a potential conflict of interest.
