# Supplementary material for: Sex Matters: Physiological Abundance of Immuno-Regulatory CD71+ Erythroid Cells Impair Immunity in Females
Source: Front Immunol. 2021 Jul 21;12:705197. doi: 10.3389/fimmu.2021.705197 (PMC8334724; doi:10.3389/fimmu.2021.705197)
Supplement: Supplementary file 1 [file DataSheet_1.pdf]

Supplementary Figure 1

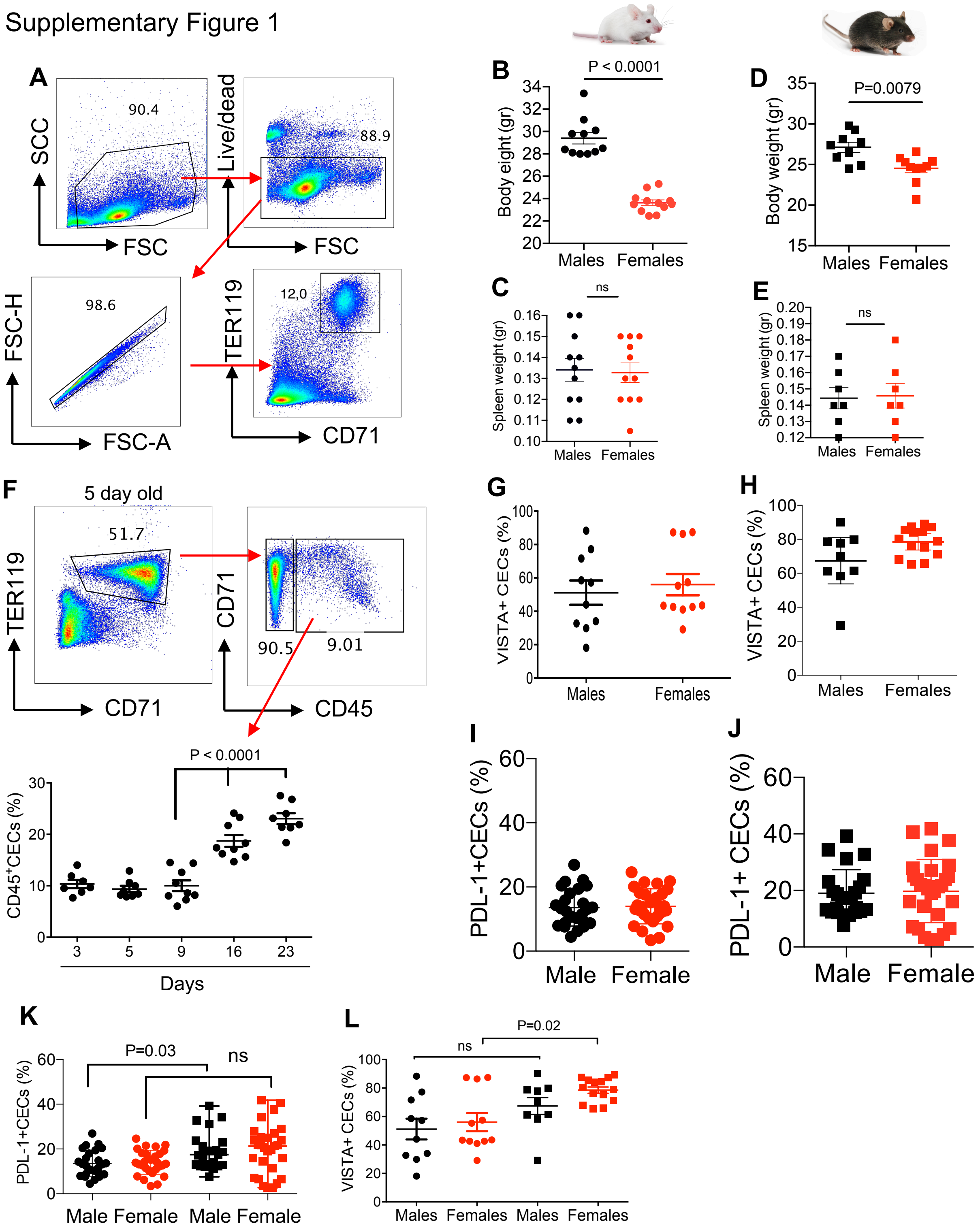

Supplementary Figure 1. (**A**) Representative flow cytometry plots of the gating strategy for CECs in a female mouse. (**B**) Cumulative data of the body weight per gram (gr) of BALB/c male versus female mice. (**C**) Cumulative data of the spleen weight in BALB/c male and female mice. (**D**) Cumulative data of the body weight per gram (gr) of C57BL/6 male versus female mice. (**E**) Cumulative data of the spleen weight in C57BL/6 male and female mice. (**F**) Representative plots and cumulative data of CD45+CECs in newborn mice. (**G**) Cumulative data of VISTA expression on CECs in male versus female BALB/c, and (**H**) C57BL/6 mice. (**I**) Cumulative data of PDL-1 expression in CECs of male versus female BALB/c, and (**J**) C57BL/6 mice. (**K**) Cumulative data comparing PDL-1 expression in male/female BALB/c with C57BL/6 mice. (**L**) Cumulative data comparing VISTA expression in male/female BALB/c with C57BL/6 mice. Each dote represents data from one individual animal and obtained from multiple independent experiments.

Supplementary Figure 2

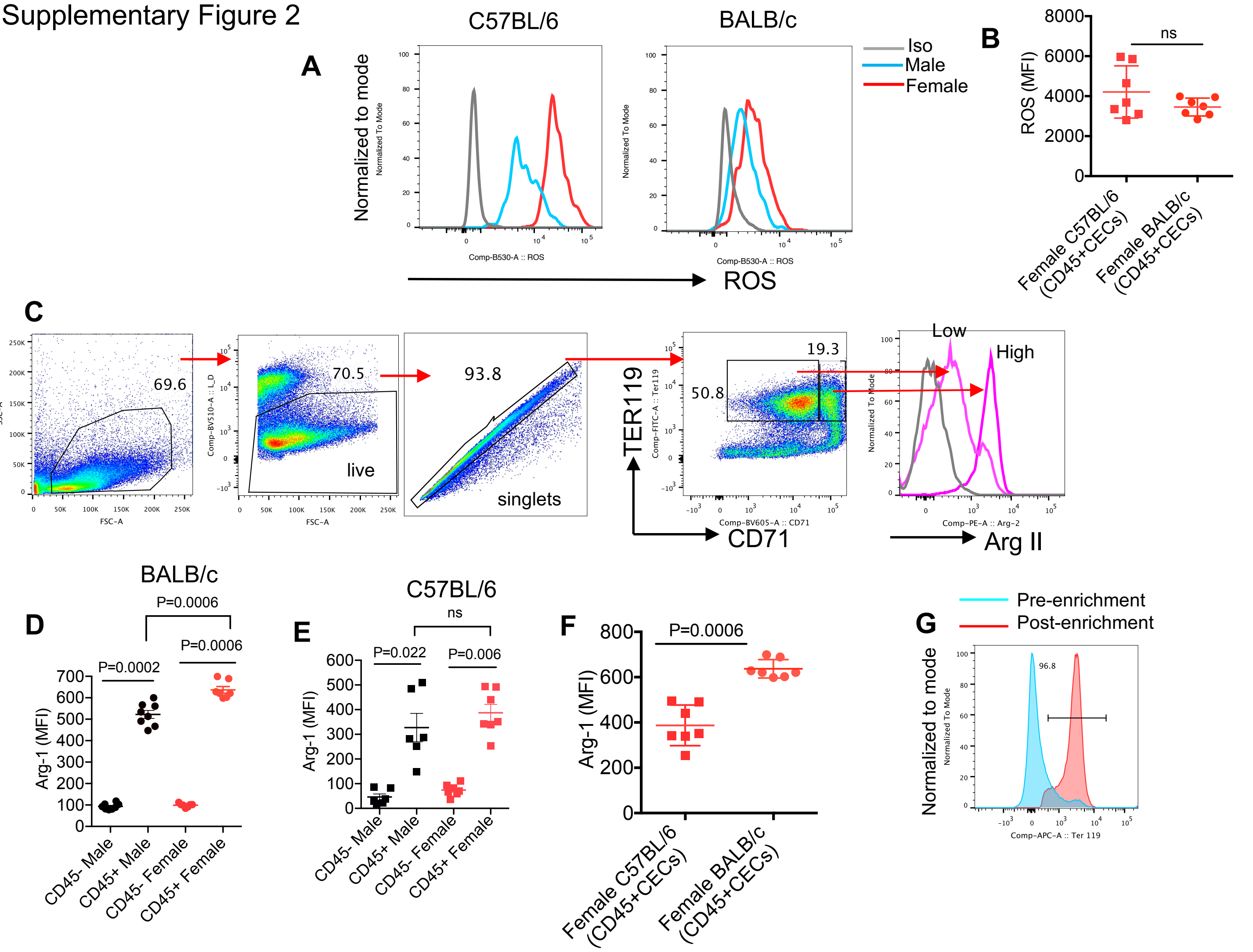

Supplementary Figure 2. (**A**) Representative histogram of ROS expression in a male versus female C57BL/6 or BALB/c mouse. (**B**) Cumulative data of the expression of ROS as measured by the mean fluorescence intensity (MFI) in CD45+CECs in female C57BL/c versus BALB/c mice. (**C**) Representative flow cytometry plots of gating strategy for the fetal liver CECs and the expression of arginase II (Arg II) in CD71^{high} and CD71^{low} CECs. (**D**) Cumulative data of arginase I (Arg I) expression in CD45- and CD45+CECs in male and female BALB/c mice. (**E**) Cumulative data of arginase I (Arg I) expression in CD45- and CD45+CECs in male and female C57BL/c mice. (**F**) Cumulative data showing expression of Arg I in CD45+CECs in female C57BL/c compred to their counterparts in female BALB/c mice. (**G**) Representative histogram of enriched CECs shown as TER119+ cells/pre and post-enrichment (purity of 96.8%).

Supplementary Figure 3

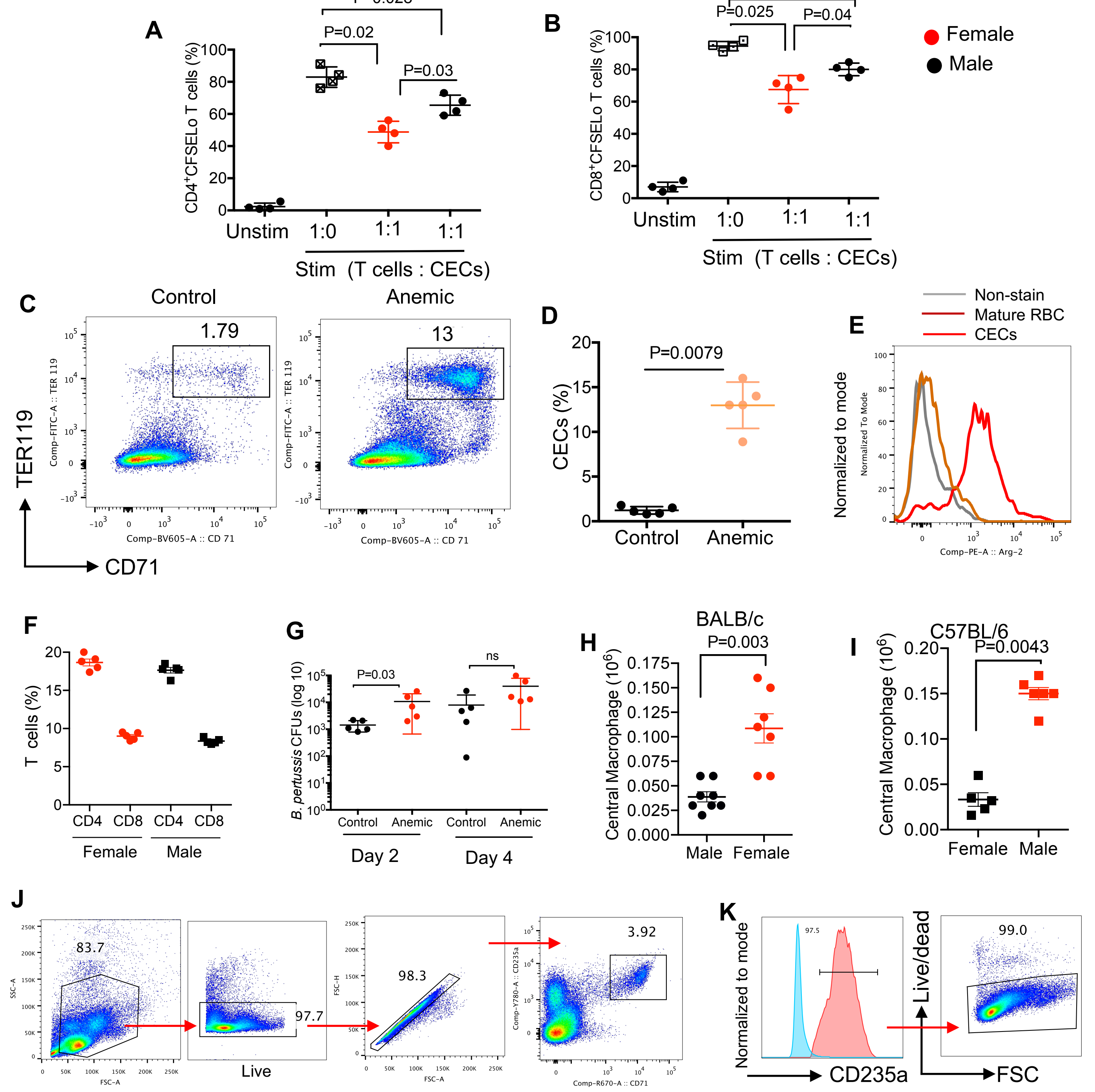

Supplementary Figure 3. (A) Cumulative data of CD4+ T cell proliferation, and (**B**) CD8+ T cells proliferation as measured by CFSE dilution in the absence or presence of CECs from female or male mice at indicated ratio of (1:1) following stimulation with anti-CD3/CD28 antibodies for 3 days. (**C**) Representative histogram of arginase II (Arg II) expression in CECs from anemic mice compared to mature red blood cells (RBCs). (**D**) Percentages of CD4+ and CD8+ T cells in the spleen of female and male C57BL/6 mice. (**E**) Representative flow cytometry plots, and (**F**) cumulative data of % CECs in the spleen of male mice following administration of the anti-TER119 antibody compared to controls (Isotype antibody-treated). (**G**) (**H**) Cumulative data showing absolute count of CD11b-CD169+F4/80+ cells in the spleen of BALB/c, and (**I**) C57BL/c female and male mice. (**J**) The gating strategy for CECs in a female human. (**K**) Representative histogram of enriched CECs shown as CD235a+ cells/pre and post-enrichment obtained from a human female. Each dote represents data from an individual animal and obtained from at least from two independent experiments.
